# Improvement of the Vietnamese Diet for Women of Reproductive Age by Micronutrient Fortification of Staples Foods and Condiments

**DOI:** 10.1371/journal.pone.0050538

**Published:** 2012-11-30

**Authors:** Arnaud Laillou, Jacques Berger, Bach Mai Le, Van Thuy Pham, Thi Hop Le, Cong Khan Nguyen, Dora Panagides, Fabian Rohner, Frank Wieringa, Regina Moench-Pfanner

**Affiliations:** 1 Global Alliance for Improved Nutrition (GAIN), Geneva, Switzerland; 2 National Institute of Nutrition (NIN), Hanoi, Vietnam; 3 Vietnam Food Administration (VFA), Hanoi, Vietnam; 4 Institut de Recherche pour le Développement (IRD), UMR Nutripass IRD-UM2-UM1, Montpellier, France; Oklahoma State University, United States of America

## Abstract

**Background:**

A micronutrient survey carried out in 2010 among randomly selected Vietnamese women in reproductive age indicated that anemia and micronutrient deficiencies are still prevalent. The objective of this study was thus to analyze the dietary micronutrient intakes of these women, to select the food vehicles to be fortified and to calculate their contributions to meet the recommended nutrient intake (RNI) for iron, zinc, vitamin A and folic acid.

**Main Findings:**

Consumption data showed that the median intake was 38.4% of the RNI for iron, 61.1% for vitamin A and 91.8% for zinc. However, more than 50% of the women had daily zinc consumption below the RNI. Rice and vegetable oil were consumed daily in significant amounts (median: 320.4 g/capita/day and 8.6 g/capita/day respectively) by over 90% of the women, making them suitable vehicles for fortification. Based on consumption data, fortified vegetable oil could contribute to an additional vitamin A intake of 27.1% of the RNI and fortified rice could increase the intake of iron by 41.4% of the RNI, zinc by 15.5% and folate by 34.1%. Other food vehicles, such as fish and soy sauces and flavoring powders, consumed respectively by 63% and 90% of the population could contribute to increase micronutrient intakes if they are properly fortified and promoted. Wheat flower was consumed by 39% of the women and by less than 20% women from the lowest socioeconomic strata.

**Conclusion:**

The fortification of edible vegetable oils with vitamin A and of rice with iron, zinc and folic acid are the most promising fortification strategies to increase micronutrient intakes of women in reproductive age in Vietnam. While rice fortification will be implemented, fortification of fish and soy sauces with iron, that has been proven to be effective, has to be supported and fortification of flavouring powders with micronutrients investigated.

## Introduction

Evidence shows that economic growth generally leads to improvements in human nutrition [1], [2], [3]. More than two decades after the “Doi Moi” (“the renovation”) that was initiated in 1986, Vietnam has achieved substantial progress in reducing poverty. Between 1998 and 2006, the national general poverty rate has fallen from 37.4% to 16.0%, and annual economic growth has stabilized at approximately 6% [4]. During this same period, the *per capita* income increased from US$300 to more than US$400 [1], [5], [6], and Vietnam significantly lowered its prevalence of child malnutrition, with a decline in stunting from 56.5% in 1990 to 29.3% in 2010 [7], [8] and in underweight from 51.5% to 17.5%. Despite this, low salary increases combined with one of the region’s highest inflation rates (which reached 11.8% in 2010 [9]) have limited the consumption of foods rich in bioavailable micronutrients, such as animal foods. Therefore, micronutrient deficiencies remain a concern.

A recent nationwide micronutrient survey conducted in 2010 highlighted that the prevalence of zinc deficiency (∼67%) and vitamin B12 deficiency (∼12%) among women of reproductive age has reached a public health problem level, according to the WHO criteria [10]. Prevalence of folate deficiency and vitamin A deficiency were considered negligible, but approximately 25% of women had a marginal folate status and 14% a marginal vitamin A status [10]. Moreover, approximately 25% of women had a ferritin concentration <30 µg/l [10], indicating low iron stores (less than 200 mg of iron). This is especially important for women of reproductive age, as iron stores of 300–500 mg before pregnancy are recommended [11].

It has been estimated that countries may lose 2–3% of their GDP if iron, iodine, and zinc deficiencies persist [12]. The prevention and control of micronutrient deficiencies are therefore a public health priority that no country can neglect. Complementary integrated intervention strategies have to be implemented (i.e. supplementation, food based approaches, dietary diversification). Amongst them, food fortification has been identified as a cost-effective and important intervention to reduce micronutrient deficiencies.

In 2000, Vietnam initiated a comprehensive approach by collaborating with international organizations to develop food fortification policies and programs. A year later, the Government issued a Strategy for Prevention and Control of Micronutrient Deficiencies in Vietnam 2001–2010. These efforts led to a Ministry of Health Decree in May 2003 that set voluntary fortification standards for a number of staple foods and condiments (complementary foods, fish sauce, wheat flour, and cooking oil). The Decree allowed many initiatives (private-public and private lead) to launch efficacy and small scale effectiveness trials to obtain evidence on fortification feasibility in Vietnam. Fish sauce was the first food vehicle studied. In a randomized controlled trial [13], rates of iron deficiency and iron deficiency anemia in 152 initially anemic women were significantly lowered in the intervention group. In a double-blind intervention with randomization by village [14], families were supplied with either fortified (5 gFe/L) or unfortified fish sauce. The effect of fortification was assessed in 576 women, and results showed a decrease in iron deficiency from 22.3% to 4.0% and a drop in the prevalence of anemia from 24.7% to 8.5% in the intervention group, whereas the control group did not show any significant change.

With the growing evidence of the impact of iron fortified fish sauce, the Global Alliance for Improved Nutrition (GAIN) supported the development of the first national plan to launch iron fortified fish sauce to prevent iron deficiency in Vietnam. Unfortunately, the privatization of the state-run fish sauce industry in the first years of the project drastically reduced the number of participating factories and the potential impact of the project. Therefore fish sauce fortification as a stand-alone intervention could not sufficiently reduce micronutrient deficiencies in Vietnam, and it became essential to conduct an analysis of the condiment (flavoring powders, sauces) and staple food (rice, wheat flour, vegetable oil) markets to examine potential additional vehicles for fortification.

A market survey in Vietnam [15] commissioned in 2009 by GAIN showed potential for a multiple food fortification strategy due to the presence of highly concentrated industries producing vegetable oil (8 producers holding 95% of the market share), fish sauces (13 producers holding 60% of the market share), flavoring powders (13 producers holding 75% of the market share), soy sauces (4 producers holding 76% of the market share) and wheat flour (8 producers holding 84% of the market share). In addition, rice fortification is currently receiving more attention because fortification technology is now available [16], [17], [18], [19], and new evidence is demonstrating the potential benefits of rice fortification to control micronutrient deficiencies [20], [21], [22], [23].

Consequently, the objective of this study was to estimate, based on measured dietary intakes, whether multiple food fortification in Vietnam has the potential to increase iron, folic acid, zinc and vitamin A daily intakes among women of reproductive age. The hypothesis was that fortification of these selected staple foods and condiments, according to the Vietnamese food fortification regulation and to the measured daily intake of these foods, will allow meeting a significant part of the daily micronutrient requirements of iron, zinc, vitamin A and folate among women of reproductive age.

## Methods

### Population Sampling

The population surveyed in the 2009 Food Consumption Survey (FCS) consisted of 7680 households, sampled from 512 clusters (104 urban and 408 rural). Households were randomly selected using a stratified 2-stage cluster sampling procedure with probability proportionate to size.

A subset of households from the FCS was randomly selected for the 2010 micronutrient survey (MNS) and was used for the analysis of the expected impact of a food fortification strategy. The sample size for 2010 MNS was estimated on the basis of a prevalence of anemia among women of reproductive age of 50%, since in the latest national anemia survey implemented in 1995, 39.9% of the non-pregnant and 52.5% of pregnant women were anemic [24]. Therefore, a sample size of 694 women per stratum (urban/rural) was calculated to get a precision of 5.0% with an expected design effect of 2.0. Anticipating an estimated 17% refusal or absence of women in the selected households, 840 households per stratum were required. Consequently a total of 56 urban and 56 rural clusters of 15 households were selected for the 2010 micronutrient study. Every woman (15–60 years old) from the selected household was surveyed. Due to limited funding, the National Institute for Nutrition first selected at random 19 provinces from the list of the total national 64 provinces. From these 19 provinces, 56 urban and 56 rural clusters were randomly chosen from those that had been selected for the 2009 FCS.

### Food Consumption Analysis

Consumption at the household level was estimated using the 24-hour recall method combined with controlled food weighing [25]. To estimate individual consumption by a woman of reproductive age (19–50 years), the energy requirement of an 18–60-y-old male was set as the reference (weight = 1) and used to weigh the specific energy requirements of other age and gender groups. A weight of 1.1 was assigned for women 15–17 years of age, 1.4 was assigned for pregnant or lactating women, and a weight of 0.9 was used for women aged 18–60 [26]. Household adult equivalent units were obtained by adding together all individual adult equivalent units in the household.

The 24-hour recall of amount of foods consumed by the household was conducted by teams of dieticians or trained personnel from the provincial medical centers. The dieticians interviewed the woman in charge of cooking the family meals. She was asked to describe household food consumption in chronological order from the time the first family members of the household woke up on the previous day until the same time on the day of the recall. To estimate the quantities of the ingredients used for preparation of the dishes as well as quantities of consumed foods and uneaten foods, various techniques were used: 1) reproduction of the quantity of food and weighing: the mother was asked to reproduce the quantity (e.g. quantity of rice cooked using the family stock) or the volume of a food (e.g. volume of fish sauce using water). The enumerator weighed these quantities using a small digital balance; 2) use of a photographic food catalogue including different serving sizes of typical dishes and calibrated cooking tools (spoon of vegetable oil, spoon of sugar, etc.); 3) estimation according to the prices of food purchases: food prices were converted into weight using market data. The woman in charge of preparing the meals was asked whether some family members ate individual meals or snacks at home or could have eaten outside the home. If this was the case, these members were interviewed directly and the amounts of those foods were included in the 24 hour recall. The dietitian then asked which members and guests took part in each of the meals. To understand the food consumption patterns of the general population, the National Institute of Nutrition (NIN) classification of food groups used for the 2000 and 2009 national consumption survey was applied, which included a wheat flour group, a flavoring powder group, a vegetable oil group and a rice group. For this article, analyses of different combinations of fortifiable food vehicles were also done to evaluate the impact of a multiple food fortification strategy using the equivalence method to calculate the mean and the median consumption among women of reproductive age, based on household overall consumption levels.

### Socioeconomic Status

Socio-economic status was calculated [27], using the Demographic Health Statistic Wealth Index to divide households surveyed into five socio-economic quintiles: the “extreme poor” (category 1), the “poor” (category 2), the “intermediate” categories 3 and 4 and the “wealthiest” (category 5). The Wealth Index was constructed from recorded data on household assets such as tables, chairs, refrigerator, air conditioners and beds and also from housing conditions (materials of house floor, house roof, main wall) and facilities (energy for cooking, electricity and latrines). Income and expenditure were not used [28].

### Fortification Levels

Fortificants and fortification levels of selected foods are presented in **Table 1**. These levels were set according to the Vietnamese standards for vitamin A in oil and to international recommendations for iron, zinc and folic acid in wheat flour (based on adult’s equivalent consumption below 75 g/day) [29] and rice (adapted from recommendation for wheat flour and based on an adult’s equivalent consumption over 300 g/day). For fish sauce the level of fortification was based on previous studies conducted in Vietnam and Thailand and taking into account the organoleptic constraints to avoid any color change or iron precipitation [30]. For the flavoring powder, the proposed fortification was set in order to not exceed the recommended maximum level of zinc fortification of 100 mg zinc/kg [31].

**Table 1:** Standards for an integrated fortification strategy.

	standards
**wheat flour** *	40 mg/kg of iron as NaFeEDTA50 mg/kg of zinc5 mg/kg of folic acid
**Rice** **	40 mg/kg of iron as micronized ferrous pyrophosphate5 mg/kg of zinc0.5 mg/kg of folic acid
**vegetable oil**	75 IU of retinyl palmitate per gram of vegetable oil
**sauces**	2.5 mg of iron as NaFeEDTA per 10 ml of sauces
**flavoring powders**	75 mg of zinc per kilogram of flavoring powders

Legend:

* based on a median consumption of less than 75 g/capita/day.

** adapted from the latest WHO recommendation on wheat and maize flour fortification not to exceed approximately 35–40% of the VRDA for any added nutrient according to a median consumption of over 300 g/capita/day.

For calculation purposes, folic acid was transformed into dietary folate equivalent (DFE): 1 µg of folic acid = 0.6 DFE; 1DFE = 1 µg food folate.

### Data Management and Statistical Analysis

The WHO recommended nutrient intake (RNI) [32] was used to assess the contribution of dietary intakes to the coverage of micronutrient daily requirements.

To calculate dietary energy and macronutrient intakes, the database of consumed food items was linked to food composition data from the Vietnamese Food Composition Database [33]. Data entry, including quality checks, was performed using SPSS software version 19™. Data management and analysis were performed with SPSS 19™. The average consumption among women consuming rice, wheat flour, sauces, vegetable oils and flavoring powders (excluding non-consumers) and the average daily intake of different micronutrients were analyzed by one-way ANOVA to compare strata and socioeconomic status. To estimate the additional intake of the micronutrient from the fortification strategy, the median consumptions for each of the selected foods (rice, wheat flour, sauces, vegetable oils and flavoring powders) were used, since the distribution of consumption was asymmetric. The median consumptions of nutrients from the fortifiable food (such as rice, wheat flour, vegetable oil and flavoring powder) by age groups were calculated by multiplying the level of fortificant (including the losses during processing and storage: 5% for iron and zinc, 30% for vitamin A and 50% for folic acid [32]) with the consumption of the different food vehicle. Iron bioavailability was estimated to be medium (10%) for diets rich in cereals but including high sources of vitamin C [32]. A low absorption of zinc was also assumed, considering the diet in Vietnam is rich in phytate [32].

### Ethical Issues

The Scientific Committees of the National Institute of Nutrition (Hanoi, Vietnam) and of the Ministry of Health (Hanoi, Vietnam) reviewed and approved the study protocol. All the women of reproductive age were informed verbally and in writing about the aims and procedures of the study. Before the enrollment, the women participating to the study signed an informed written consent. On behalf of the minors participating to the study, the parents signed the informed written consent for their involvement.

## Results

The food consumption pattern of the women from the 1503 selected households was analyzed, of which 49.0% were urban and 51.0% were rural. Among these households, 16.2% were classified in the “extreme poor” category (category 1), 15.1% in the “poor” category (category 2), 18.3% in intermediate number 3 category, 21.2% in intermediate number 4 category, and 29.3% in the “wealthiest” category (category 5).

**Figure 1:**
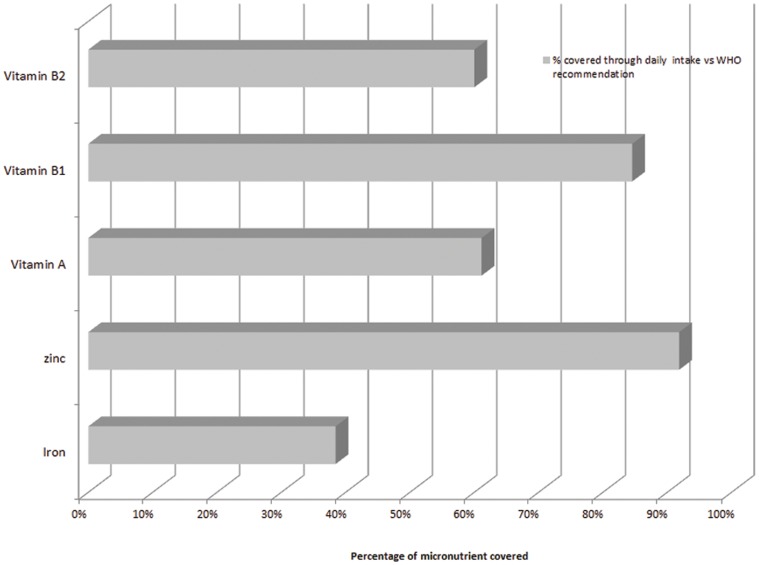
Percentage of retinol, zinc, vitamin B1 and B2 and iron requirement covered through the daily median intake of a woman of reproductive age.

The mean energy intake was 1944 Kcal/day, with a significantly higher quantity of carbohydrates consumed in rural versus urban areas (p<0.001) and a significantly higher quantity of lipids consumed in urban versus rural areas (p<0.001) (**Table 2**). No significant difference was observed in mean iron, vitamins A (as retinol activity equivalent) and B_1_ consumption between the urban and rural populations, while significant higher daily intakes of zinc and significant lower intake of vitamin B_2_ was noted in the rural population. As shown in **Figure 1**, the median intake of a woman covered 38% of the iron RNI, 92% of the zinc RNI, 85% of the vitamin B1 RNI, 60% of the vitamin B2 RNI and 61% of the vitamin A WHO RNI. The Vietnamese food composition table does not provide information on other nutrients such as vitamin B12 and folate to generate a more complete overview of gaps in micronutrient intake in vulnerable groups. In the bottom tertile of consumers, micronutrient coverage of RNI were 33% for iron, 80% for zinc, 73% for vitamin B1, 46% for vitamin B2, 38% for vitamin A.

**Table 2:** Food intake and food balance characteristics^1^ by rural and urban residence.

	n	Energy (Kcal)	Protein (g)	Carbohydrates (g)	Lipid(g)	Iron (mg)	Zinc (mg)	Vitamin A (µg retinol activity equivalent)	Vitamin B1 (mg)	Vitamin B2 (mg)
*Total*	1497	1944.5±23.1/1802.9	74.8±0.9/69.5	323.4±4.1/297.6	39.3±0.9/32.3	12.8±0.4/11.3	9.8±0.1/9.0	391.9±11.6/305.6	1.06±0.01/0.93	0.75±0.01/0.66
*Areas*										
** Rural**	761	1989.0±34.4/1814.0	73.3±1.4/66.1	342.9±6.6/312.0	36.0±1.0/30.6	13.1±0.8/11.3	10.2±0.2/9.1	383.4±16.9/289.5	1.03±0.02/0.89	0.71±0.02/0.63
** Urban**	736	1898.6±30.7/1781.2	76.3±1.2/72.4	303.1±4.5/279.9	42.7±1.5/34.7	12.4±0.2/11.2	9.5±0.1/9.0	400.8±15.7/313.7	1.08±0.02/0.97	0.79±0.02/0.69
** P** 2		P = 0.051	P = 0.098	p<0.001	p<0.001	P = 0.380	p<0.01	P = 0.454	P = 0.09	p<0.01

1 mean ± standard of error of the mean/median for women consuming the food.

2 ANOVA test on the mean.

Rice, vegetable oil and flavoring powders were consumed daily by almost the entire population whereas fish and soy sauces were consumed by two thirds and wheat flour by only two fifths of the women (**Table 3**). The percent of women consuming rice was very similar throughout the different socioeconomic categories and residence areas (but significantly different, p<0.0001) whereas the consumption of vegetable oil, flavoring powders, wheat flour and sauces tended to increase with socioeconomic status and was significantly higher (p<0.001) in urban compared to rural populations.

**Table 3:** Percentage of Vietnamese households consuming selected foods daily, by socioeconomic status (quintile) and residence.

	n	Rice	Vegetable oil	Flavoring powders	Sauces	Wheat flour
*Socioeconomic status*						
** 1**	243	99.6	80.7	87.2	31.3	19.3
** 2**	226	100	91.6	93.8	53.5	33.6
** 3**	274	99.3	94.5	94.2	63.5	41.2
** 4**	318	100	93.7	90.6	72.6	42.8
** 5**	440	100	94.3	92.5	77.3	47.7
** p**		p<0.0001	p<0.0001	p<0.05	p<0.0001	p<0.0001
*Areas*						
** Rural**	766	99.6	89.4	89.3	56.3	31.1
** Urban**	737	100	93.9	94.3	69.6	46.7
		p<0.01	p<0.0001	p<0.0001	p<0.0001	p<0.0001
**Total population**	1503	99.8	91.5	91.7	62.8	38.8

Legend: Socio-economic categories: 1: the “extreme poor”; 2: the” poor”, 3 and 4: the “intermediate” and 5: the “wealthiest”; socioeconomic status for 2 households are missing.

The mean and median daily intakes of the selected foods among women who consumed these foods are presented in **Table 4**, according to socioeconomic groups and residence areas. The mean±SD (and median) daily intake was 347.2 g±4.2 g (320.4 g) for rice, 44.0±1.9 g (33.8 g) for wheat flour, 11.1 g±0.3 g (8.6 g) for vegetable oil, 10.6 g±0.7 g (6.8 g) for sauces, and 4.9 g±0.2 g (3.3 g) for flavoring powders. Whereas daily intake of oil, sauces and flavoring powders did not differ between socioeconomic categories and areas of residence, significant higher quantities of rice and lower quantities of wheat flour were consumed by women living in rural areas compared to urban areas. Also, the wealthiest population group consumed significantly more wheat flour than the poorest group. For rice, the opposite was observed with the poorest consuming more rice than the wealthiest.

**Table 4:** Mean±SEM and median^1^ (g) consumption per woman of reproductive age equivalent collected through the 24 hour recall food intake among households, by socioeconomic status and residence (consumers only considered).

	n	Rice	Wheat flour	Vegetable oil	Sauces^2^	Flavoring powders
*Socioeconomic status*						
** 1**	243	389.9±11.0/350.6	30.6±4.9/21.6	12.1±0.9/7.5	14.5±3.3/6.4	4.8±0.4/3.3
** 2**	226	371.8±8.8/349.0	32.6±2.6/29.3	10.7±0.6/8.6	8.4±0.9/6.3	4.6±0.4/2.9
** 3**	274	345.3±8.5/320.6	38.7±2.6/32.4	11.9±0.7/8.3	10.1±1.0/6.8	4.9±0.5/2.8
** 4**	318	351.5±13.1/308.3	40.9±2.3/37.2	11.0±0.5/8.8	11.3±2.2/6.3	4.5±0.2/3.3
** 5**	440	308.9±5.1/292.2	56.0±4.7/37.4	10.4±0.4/9.5	10.3±0.5/7.7	5.4±0.3/4.1
** P** 3		P<0.001	P<0.01	ns	ns	ns
*Areas*						
** Rural**	766	371.4±7.1/337.5	37.7±1.8/33.8	11.6±0.4/8.6	10.4±0.7/6.5	5.0±0.2/3.2
** Urban**	737	322.2±4.3/308.0	48.4±3.0/36.0	10.6±0.3/8.6	10.8±1.0/6.9	4.8±0.2/3.6
** P** 3		P<0.001	P<0.001	ns	ns	ns

1 mean ± standard of error of the mean/median for women consuming the food.

2 include only fish and soya sauces and considering 87% of the sauces consumed are fish and soy sauces.

3 ANOVA test on the mean.

Legend: Socio-economic categories: 1: the “extreme poor”; 2: the” poor”, 3 and 4: the “intermediate” and 5: the “wealthiest» and socioeconomic status for 2 households are missing.

**Figure 2:**
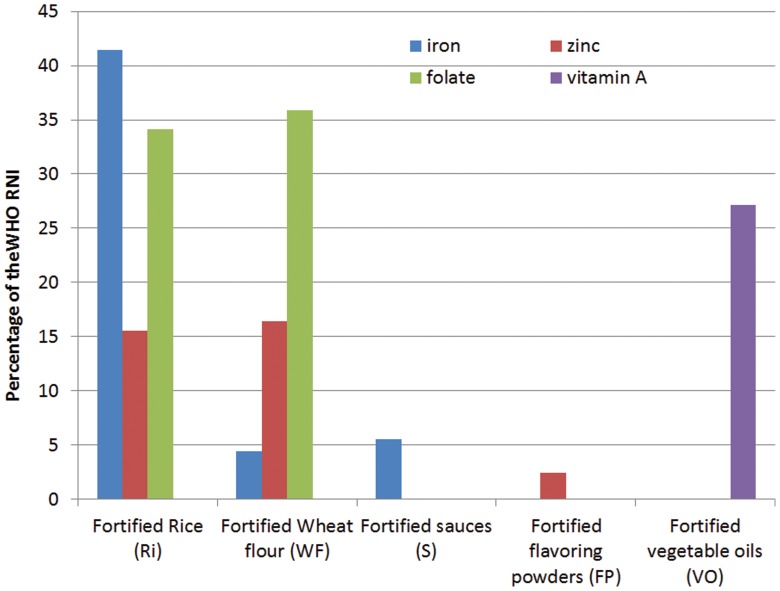
Percentage of the WHO RNI for women of reproductive age potentially provided through different food vehicles for women who consume them.

Based on food consumption data and median level of consumption, the amounts of iron, folate, zinc and vitamin A provided by the individual food vehicles after fortification to women of reproductive age were calculated (**Table 5** **)**. Fortified rice could provide 15.5% of the WHO RNI for zinc, 34.1% for folate, and 41.4% for iron (**Figure 2**). Wheat flour could provide 16.4% of RNI for zinc and 35.9% for folate but less than 5% for iron. Sauces could contribute 5.5% of iron RNI and flavoring powders less than 2.4% of zinc RNI.

**Figure 3:**
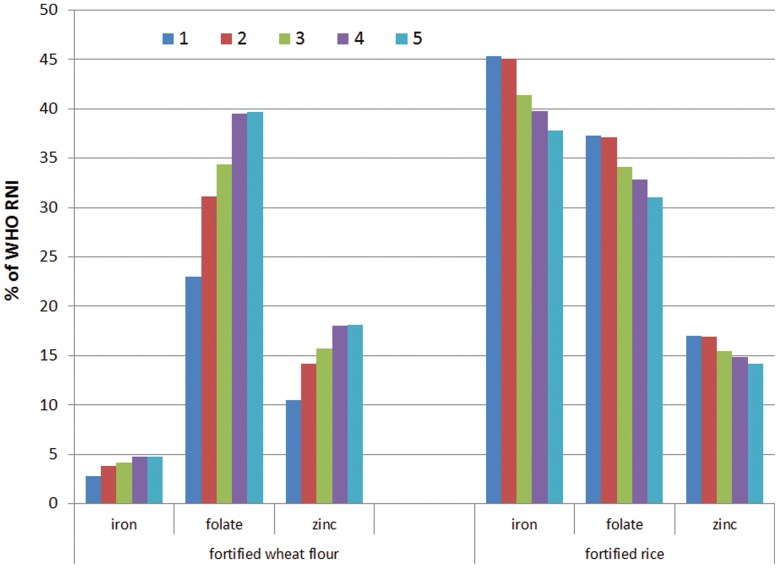
Percentage of the WHO RNI for women of reproductive age potentially provided through consumption of fortified wheat flour and rice among socioeconomic groups. Socio-economic categories: 1: the “extreme poor”; 2: the “poor”, 3 and 4: the “intermediate” and 5: the “wealthiest”, and socioeconomic status for 2 households are missing.

As the consumption of rice and wheat flour was significantly different among socioeconomic groups, we calculated the contribution of fortification to the WHO RNI provided to each group amongst those who consumed these foods (**Figure 3**). The figure clearly shows that the poorest part of the women will benefit more from rice fortification while the wealthiest group of women will benefit more from both fortifications. For instance, considering the extreme poor category, fortified rice could provide 45.3% of RNI for iron, 17% for zinc and 37.3% for folate, while fortified wheat flour could provide 3% of WHO RNI for iron, 23% for folate and 10.5% for zinc.

**Table 5:** Estimated daily contribution from fortified foods (using median amounts consumed per day by women, and proposed fortification levels)^1,2^.

	Nutrient contribution per women equivalent unit from other fortification			
	Iron (mg/d)	Zinc (mg/d)	Folate (µg/d)	Retinyl palmitate (µg RE/d)
**Fortified Rice**	12.2 (9.6–15.3)	1.5 (1.2–1.9)	136 (108–171)	–
**Fortified Wheat flour (WF)**	1.3 (0.7–2.1)	1.6 (0.9–2.6)	144 (77–232)	–
**Fortified sauces (S)**	1.6 (1.0–2.9)	–	–	–
**Fortified flavoring powders (FP)**	–	0.2 (0.1–0.4)	–	–
**Fortified vegetable oils (VO)**	–	–	–	135.5 (81.9–222.1)

1 Values are medians (25^th^ and 75^th^ percentiles).

2 assuming 5% of losses for iron, 30% of losses for retinyl palmitate and 5% of losses for zinc.

## Discussion

The analysis of the food consumption survey shows that the daily diet did not provide sufficient amounts of micronutrients such as iron and vitamin A for women in reproductive age. Indeed, based on the median intake, diets contributed to only approximately one third of iron requirements (WHO RNI, [32]), and two thirds of vitamin A and B2 requirements, and more than 80% of zinc and vitamin B1 requirements. This support that integrated strategies to increase the micronutrient intake of women in reproductive age have to be considered. Amongst them, food fortification has been identified as a cost-effective and important intervention to reduce micronutrient deficiencies [12], [34] and is under consideration by the Vietnamese government.

The study also shows that rice, vegetable oil and flavoring powders were widespread, with more than 90% of women of reproductive age consuming them daily. This make these foods relevant food vehicles for micronutrient fortification [32].

As shown by the calculations based on fortification levels of these food vehicles and their consumption by women, fortification of oil with vitamin A will significantly contribute to increasing the intake of vitamin A by one fourth of the RNI. The process of fortifying vegetable oil with vitamin A is well established [35]. Assuring an adequate vitamin A status for women before and during pregnancy and lactation is essential and 14% of the Vietnamese women in reproductive age had a marginal vitamin A status [10]. Moreover, it is recognized that during lactation, maternal status or intake of vitamin A strongly affects the amount of vitamin A in breast milk [36]. Therefore increasing vitamin A status of women should have an impact on infants and young children which are especially at risk for vitamin A deficiency as demonstrated in the recent study indicating a marginal vitamin A status in half of Vietnamese children [10]. Increasing vitamin A intake of women is particularly crucial as WHO no longer recommends vitamin A supplementation post-partum as a public health intervention for the prevention of maternal and infant morbidity and mortality [37]. In addition, according to the micronutrient study we conducted (personal communication [38]), more than 50% of the women were vitamin D deficient (below 50 nmol/L [39]) and more than 30% had insufficient (circulating 25(OH)D between 51–74 nmol/L) vitamin D status [39]. Thus the addition of vitamin D to vegetable oil should be considered if the high prevalence of vitamin D is confirmed as a public health problem in Vietnam.

Rice was consumed daily by all women with a median consumption exceeding 300 g per day. Fortifying rice with iron, zinc and folic acid as proposed will provide 40% of daily iron requirements and 30% of folate requirements. This is particularly important for women in reproductive age that are planning on having a baby. Indeed, several studies have shown that iron stores at conception are a strong predictor of maternal iron status and risk of anemia later in pregnancy [40], [41]. Moreover, it is very difficult to replenish depleted iron stores once pregnant, particularly for poor women who have less access to and less knowledge of supplements. An adequate folate status is also very important for women of reproductive age especially before conception and during early pregnancy to prevent the risk of neural tube defects (NTD) [36]. Our study carried out in 2010 shows that half of these Vietnamese women have low iron stores and the same proportion a marginal folate status [10]. Another recent study among women living in Hanoi and Hai Phuong Provinces indicated that 60% had a suboptimal folate status [42]. These results support the relevance to increase their iron and folate intakes. Fortification of rice has been proven to be effective in improving iron status for children between 6 to 13 years of age in India when fortified rice provides 17 to 19 mg iron of micronized ferrous pyrophosphate daily [23], [43]. Fortification of rice with folic acid in Vietnam would provide 136 µg/day of folate to women. In North America, daily intake of 140 µg folic acid from fortified wheat flour, corresponding to 233 µg folate, was shown to have a similar impact on folate status as daily supplementation with 400 µg folic acid (667 µg folate), when taken during the preconception period and given over a sufficient period of time [44]. Moreover, it has been demonstrated in South Africa and the United States that large scale fortification of wheat flour with folic acid prevents neural tube defects [45], [46].

Regarding zinc, dietary intake data indicated that despite zinc intakes covering approximately 80% of zinc RNI, more than 50% of women had a zinc intake lower than 9 mg/day which is below WHO RNI of 9.8 mg/day. This would explain the high prevalence of zinc deficiency in the Vietnamese women (67%) based on measurement of plasma zinc [10]. Moreover zinc intakes were calculated assuming a bioavailability of less than 30% (low bioavailability) [47]. However, the Vietnamese diet is based on rice, green vegetables, white meat (pork and chicken) and green tea and consequently rich in phytates and polyphenols. Consumption of red meat, where zinc is the most bioavailable, is low [48]. This would suggest that the bioavailability of zinc from traditional Vietnamese diet was probably lower than expected.

Rice fortification would provide 15% of zinc requirements and flavoring powder only 2%. In Thailand, seasoning powder fortified with zinc (5 mg), iron (5 mg), vitamin A (270 µg), and iodine (50 µg) per serving enhanced levels of hemoglobin, zinc, and iodine in 569 preschoolers and adolescents [49]. This shows that fortification standards of flavoring powder in Vietnam could be revised by considering adding other micronutrients and increasing their contents taking into account the cost and potential organoleptic issues. Moreover, an impact study has to be carried out before any decision can be taken. Increasing the zinc fortification of rice could also be considered.

Fish and soy sauces were consumed by one third of the wealthiest women and by two third of the women in the lowest two socioeconomic categories. Iron fortification of fish sauce (13) and soy sauce [50] have been proven to be efficient in women in randomized controlled trials. In Vietnam, iron deficiency and anemia were significantly decreased and almost disappeared in approximately one year by the regular consumption of fortified fish sauce (5 gFe/L) freely used for traditional cooking and eating practices [14]. The current standards for fish sauce fortification recommend a dose of 2.5 mg iron as NaFeEDTA to avoid coloration and iron precipitation [30]. This lower quantity may decrease the impact on iron status but NaFeEDTA is known to enhance the absorption of both the intrinsic food iron [51] and iron used in other fortified food and may have a beneficial effect on overall iron absorption from the diet. Since sauces are often consumed with rice and vegetables, fortified sauces may support additional iron absorption from these foods.

Wheat flour is widely fortified around the world with multiple micronutrients such as iron, zinc and folic acid and could provide, in the case of Vietnam, 36% of the RNI for folate and 16% for zinc but only 4% for iron in women who consume wheat flour. Unfortunately wheat flour consumption is not widespread in Vietnam, with only 19% of the extreme poor population and 39% of women consuming wheat flour regardless the socioeconomic groups, and therefore limiting the impact of wheat flour fortification nationwide.

The risk of excess intake from a multiple food fortification strategy depends on the combination consumed daily and varies with the micronutrient. More than 55% of households were consuming the following combinations on a daily basis: vegetable oil-sauces-flavoring powder (56.6%), vegetable oil-sauces (60.2%), vegetable oil-flavoring powder (84.8%) or vegetable oil-rice (91.5%). Only about one fourth of the population was found to consume wheat flour with another food. If fortified rice is distributed through the open market, for women who consume all food vehicles at the 75^th^ percentile level, the five food vehicles could provide 68.9% of the WHO RNI for iron, 100.8% of the WHO RNI for folic acid, 50.4% of the WHO RNI for zinc and 44.4% of the WHO RNI for vitamin A. The tolerable upper limits (UL) for nutrients are the highest level of nutrient intake at which no adverse effects occur. According to The United States Food and Nutrition Board of the Institute of Medicine the UL for zinc is 40 mg, 45 mg for iron, 3,000 µg retinol equivalent for vitamin A and 1,000 µg for folate for adults [32]. Even when all above mentioned food vehicles are fortified and the women are eating as described in table 2, the median consumption of 12.3 mg of zinc, 26.4 mg of iron, 441.4 µg vitamin A as retinol activity equivalent (from diet and fortification) and 280 µg folate per day for a woman (from fortification) are all well below the upper limits. Those intakes also stay under the UL for a woman who consumes all the above fortified foods at the 75^th^ percentile level (13.9 mg of zinc, 31.6 mg of iron, 528 µg vitamin A as retinol activity equivalent (from diet and fortification) and 403 µg folate per day for a woman (from fortification).

Finally, this fortification strategy has little probability of impacting negatively the populations living in mountainous areas, where malaria issues could remain. For instance, we have demonstrated that based on a median intake of these fortified foods, Vietnamese children less than 5 years of age will ingest from 4.7 mg to 9.9 mg of iron per day depending on the age group [52]. Those doses are not considered as a high iron dose which could stimulate the growth of pathogenic bacteria [53]. Moreover, a recent Cochrane review [54] concluded that routine iron supplementation should not be withheld from children living in countries where malaria is prevalent when regular malaria surveillance and health services were available (the case in Vietnam). Therefore, fortification should not be seen as threat.

### Conclusion

This study shows that Vietnamese women of reproductive age had a diet that did not allow meeting recommended dietary requirements for iron, zinc and vitamin A. However, preventing iron, vitamin A and other micronutrient deficiencies is crucial especially during periods of high rice and/or food prices and when less money is spent on vegetables, fruits, dairy products, eggs and meat [55], leading to a shift away from preformed vitamin A [56], heme iron, zinc and folic acid rich products. Based on consumption of staple foods and condiments by women in reproductive age, it can be concluded that fortification of oil with vitamin A would improve their vitamin A status. Fortification of rice and wheat flour with iron, zinc and folate could significantly contribute to increase the intakes of these micronutrients. Rice fortification is the most promising action as rice is daily consumed by the Vietnamese population but it may require a couple of months or even years before its implementation at the national level. Currently, strategies aiming at introducing fortified rice through village rice millers are being piloted. On the other hand, the impact of fortifying wheat flour would be probably limited due to its low consumption. Meanwhile, aside from rice fortification, fortification of fish and soy sauces with iron, that has been proved to be efficient, has to be sustained and the feasibility of fortifying flavoring powders with several micronutrients needs to be investigated.
